# Plasma neuregulin 1 as a synaptic biomarker in Alzheimer’s disease: a discovery cohort study

**DOI:** 10.1186/s13195-022-01014-7

**Published:** 2022-05-23

**Authors:** Agathe Vrillon, François Mouton-Liger, Matthieu Martinet, Emmanuel Cognat, Claire Hourregue, Julien Dumurgier, Elodie Bouaziz-Amar, Ann Brinkmalm, Kaj Blennow, Henrik Zetterberg, Jacques Hugon, Claire Paquet

**Affiliations:** 1 Université Paris Cité, Inserm U1144, Paris, France; 2 Université Paris Cité, Center of Cognitive Neurology, Lariboisière Fernand-Widal Hospital, APHP, 200 rue du Faubourg Saint-Denis, 75010 Paris, France; 3 Université Paris Cité, Department of Biochemistry, APHP GHU Nord Lariboisière-Fernand Widal, Paris, France; 4 Institute of Neuroscience and Physiology, The Salhgrenska Academy at the University of Gothenburg, Mölndal, Sweden; 5 Clinical Neurochemistry Laboratory, Sahlgrenska University Hospital, Mölndal, Sweden; 6 UK Dementia Research Institute at UCL, London, UK; 7 Department of Neurodegenerative Disease, UCL Institute of Neurology, London, UK; 8 Hong Kong Center for Neurodegenerative Diseases, Hong Kong, China

**Keywords:** Alzheimer’s disease, NRG1, Synaptic pathology, Plasma biomarker

## Abstract

**Background:**

Synaptic dysfunction is an early core feature of Alzheimer’s disease (AD), closely associated with cognitive symptoms. Neuregulin 1 (NRG1) is a growth and differentiation factor with a key role in the development and maintenance of synaptic transmission. Previous reports have shown that changes in cerebrospinal fluid (CSF) NRG1 concentration are associated with cognitive status and biomarker evidence of AD pathology. Plasma biomarkers reflecting synaptic impairment would be of great clinical interest.

**Objective:**

To measure plasma NRG1 concentration in AD patients in comparison with other neurodegenerative disorders and neurological controls (NC) and to study its association with cerebrospinal fluid (CSF) core AD and synaptic biomarkers.

**Methods:**

This retrospective study enrolled 127 participants including patients with AD at mild cognitive impairment stage (AD-MCI, *n* = 27) and at dementia stage (*n* = 35), non-AD dementia (*n* = 26, Aβ-negative), non-AD MCI (*n* = 19), and neurological controls (*n*=20). Plasma and CSF NRG1, as well as CSF core AD biomarkers (Aβ 42/Aβ 40 ratio, phospho-tau, and total tau), were measured using ELISA. CSF synaptic markers were measured using ELISA for GAP-43 and neurogranin and through immunoprecipitation mass spectrometry for SNAP-25.

**Results:**

Plasma NRG1 concentration was higher in AD-MCI and AD dementia patients compared with neurological controls (respectively *P =* 0.005 and *P* < 0.001). Plasma NRG1 differentiated AD MCI patients from neurological controls with an area under the curve of 88.3%, and AD dementia patients from NC with an area under the curve of 87.3%. Plasma NRG1 correlated with CSF NRG1 (*β* = 0.372, *P* = 0.0056, adjusted on age and sex). Plasma NRG1 was associated with AD CSF core biomarkers in the whole cohort and in Aβ-positive patients (*β* = −0.197–0.423). Plasma NRG1 correlated with CSF GAP-43, neurogranin, and SNAP-25 (*β* = 0.278–0.355). Plasma NRG1 concentration correlated inversely with MMSE in the whole cohort and in Aβ-positive patients (all, *β* = −0.188, *P* = 0.038; Aβ+: *β* = −0.255, *P* = 0.038).

**Conclusion:**

Plasma NRG1 concentration is increased in AD patients and correlates with CSF core AD and synaptic biomarkers and cognitive status. Thus, plasma NRG1 is a promising non-invasive biomarker to monitor synaptic impairment in AD.

**Supplementary Information:**

The online version contains supplementary material available at 10.1186/s13195-022-01014-7.

## Introduction

Synaptic impairment is a core feature of Alzheimer’s disease (AD) and one of the earliest detectable changes [1, 2]. Neuropathological examination has demonstrated that synaptic demise shows a higher association to cognitive decline than amyloid plaque load or neurofibrillary tangle pathology [3, 4]. Positron emission tomography (PET) imaging using synaptic tracers indicates that synaptic density is significantly reduced in the hippocampus in AD patients, especially in its early symptomatic stages [5, 6]. The evaluation of several synaptic proteins has been achieved in the cerebrospinal fluid (CSF) [7–10]. Presynaptic synaptosomal-associated protein 25 (SNAP-25), synaptotagmin, or growth-associated protein-43 (GAP-43) as well as post-synaptic neurogranin levels are altered in AD CSF and are reliable biomarkers of synaptic impairment, as early as in the preclinical stage of the disease [11, 12]. Those synaptic biomarkers also contribute to the understanding of the underlying pathological processes of the cognitive decline. Alteration of current CSF synaptic biomarkers appears to show specificity to AD, indicating AD as the pathology with the highest synaptic involvement [11, 12]. Moreover, the different synaptic proteins involved in various processes in pre- and post-synaptic compartments most likely reflect different mechanisms at play at the synapse [13]. Thus, synaptic biomarker investigation appears as a key tool to investigate the pathological mechanisms responsible for synaptic damage.

Regarding blood, the presynaptic betasynuclein measured using quantitative mass spectrometry could discriminate AD and CJD from controls and other neurodegenerative disorders [14]. Other synaptic markers have been explored in blood but so far, due to the existence of peripheral expression or of other factors of variability, there is no validated reliable biomarker of synaptic pathology [13]. Plasma markers allow for easy, cost-effective, and repeated measurements both in research and in clinical settings. Synaptic impairment markers are a category of biomarkers expected to be most closely correlated with cognitive function. It would make a synaptic plasma biomarker of high interest for monitoring AD progression, as well as for screening and inclusion, and measure of the therapeutic response in clinical trials.

Neuregulin 1 (NRG1), a protein encoded by the *NRG1* gene, is a member of the epithelial growth factor (EGF) family. They constitute ligands with a high affinity for ErbB tyrosine kinase receptors. NRG1 is implicated in many processes during neural development including the proliferation of neuronal progenitors, neuron migration and survival, axon guidance, glial development, and myelination, as well as synaptogenesis [15, 16]. In the adult brain, NRG1 is expressed in multiple regions and regulates neurotransmission and synaptic plasticity [17]. Membrane-bound, NRG1 requires processing by a protease to initiate release and signaling. Among implicated proteases, NRG1 can undergo cleavage by the β-site amyloid precursor protein cleaving enzyme 1 (BACE1) at multiple sites [18–20]. Proteolytic processing results in the secretion of soluble forms that will further activate ErbB receptors, mainly at the post-synaptic level. NRG1 and its receptor ErbB4 levels have been found altered in the human AD brain, both in the hippocampus and cortex [21, 22]. In CSF, two studies including our prior work have reported modified NRG1 levels in AD patients compared with controls and to patients with non-AD-related cognitive decline [23, 24].

The purpose of our study was to investigate plasma NRG1 levels in a cohort of patients with cognitive decline due to AD, non-AD-related cognitive decline, and neurological controls and to assess its association with core AD CSF biomarkers, CSF synaptic markers, and cognitive status.

## Methods

### Cohort

A total of 127 patients from the Cognitive Neurology Center, Lariboisière Fernand Widal Hospital, Université Paris Cité, was retrospectively included in our study comprising patients with AD at the stage of mild cognitive impairment (AD-MCI, *n* = 27) and at the stage of dementia (*n* = 35), non-AD-related mild cognitive decline (non-AD MCI, *n* = 19), non-AD dementia (*n* = 26), and neurological controls (NC, *n* = 20).

Patients had undergone CSF biomarker analysis from 2012 to 2015 including Aβ 42/Aβ 40 ratio, tau phosphorylated on threonine 181 (p-tau), and total-tau (t-tau) measurements, in the context of the diagnostic workup of a cognitive complaint. Consensus diagnoses were made by neurologists, geriatricians, neuropsychologists, neuroradiologists, and biologists after comprehensive neurological examination, neuropsychological assessment, brain imaging, and CSF biomarker analysis, according to current diagnostic criteria [25–29].

All AD patients met the NIA-AA research framework criteria and displayed a CSF profile on the AD *continuum* [26]. AD-MCI patients followed Albert et al. definition of MCI due to AD [25]. The non-AD MCI group comprised subjects with cognitive decline unrelated to AD, encompassing diagnosis of psychiatric disorders, systemic disorders, or non-neurodegenerative disorders. The non-AD dementia group included patients with dementia with Lewy bodies (DLB, *n* = 6), behavioral variant frontotemporal dementia (FTD, *n* = 9), and vascular dementia (VD, *n* = 7). Non-AD MCI and non-AD dementia patients had normal amyloid ratio Aβ 42/40 and normal or abnormal p-tau and t-tau. NC included patients with subjective cognitive decline, anxiety, depression, or sleep apnea syndrome, presenting with normative or sub-normative cognitive scores, normal CSF biomarkers, and an absence of cognitive decline at follow-up.

This study was approved by the Bichat Hospital Ethics Committee of Paris Diderot University (N°10–037, 18/03/2010) and all the participants have given their written consent.

### CSF biomarkers

CSF was obtained through a lumbar puncture; the second and third milliliters were collected and centrifuged to prevent blood contamination. The supernatant was stored at − 80 °C until further analysis.

CSF core AD biomarkers (Aβ 42, Aβ 40, p-tau, and t-tau) were analyzed at the Department of Biochemistry at Lariboisiere University Hospital Paris, France, using commercially available INNOTEST® kits (Fujirebio Europe NV, Gent, Belgium) in a delay of 1 month after collection. CSF profiles were analyzed according to the following cut-offs: A+: Aβ42/Aβ40 ratio < 0.076; T+: p-tau > 58 pg/mL; N+: t-tau > 340 pg/mL [26]. Patients were classified as Aβ-positive and Aβ-negative according to the Aβ42/Aβ40 ratio.

CSF NRG1 concentration was measured using the Human NRG1 DuoSet ELISA kit (R&D Systems, Minneapolis, MN) as reported in Mouton-Liger et al. [24].

All the CSF synaptic markers were assessed at the Clinical Neurochemistry Laboratory at the Sahlgrenska University Hospital (Mölndal, Sweden). CSF neurogranin and CSF GAP-43 concentrations were measured using in-house developed ELISAs [8, 10]. CSF SNAP-25 concentration was measured by immunoprecipitation mass spectrometry according to a validated method [9, 11].

### Plasma NRG1 measurement

Blood samples were obtained through venipuncture under fasting condition and collected into ethylenediaminetetraacetic acid (EDTA) tubes. Samples were centrifuged at 2000×*g* for 20 min at 4°C. Plasma supernatant was collected and frozen at −80°C until further use. Prior to analysis, samples were centrifuged at 2000*g* for 10 min after thawing at room temperature. Plasma NRG1 was assessed using the Human NRG1 DuoSet ELISA kit (R&D Systems) in Mölndal, Sweden, following the manufacturer’s protocol. This assay has been shown to be highly sensitive to human NRG1 alpha-subunit with a sensitivity of 125–4000 pg/mL [24, 30, 31]. Plasma samples from study participants were analyzed in duplicates. Intra-plate and inter-plate coefficients of variation were respectively 5.9% and 7.4%. Ten samples (7.9% of samples total) were below the detection limit of the assays, including 4 NC, 3 AD, 2 non-AD dementia, and one non-AD MCI other patient. For those samples, plasma NRG1 levels were interpolated from the standard curve or if this was not possible due to the very low signal the values were imputed to the lowest interpolated value. One outlier sample (plasma NRG1 value > mean+5SD) was excluded from the analysis.

### Statistical analysis

Participants’ characteristics were examined in 5 groups: NC, AD-MCI patients, AD dementia, non-AD MCI, and non-AD dementia. Patients were also divided into Aβ-positive and Aβ-negative groups according to their CSF Aβ42/Aβ40 ratio using the clinically validated cut-off. Data are expressed as mean (standard deviation) for continuous variables or percentage (%) for categorical variables. We used the Kruskal-Wallis test to compare age and Mini-Mental State Examination (MMSE) scores between groups and Pearson’s chi-square for sex. Fluid biomarker levels were log-transformed prior to analysis and compared using a one-way ANCOVA adjusted on age and sex followed by a post hoc least significant difference (LSD) test for pairwise group comparisons, adjusted for multiple comparisons (Bonferroni). Delay between sample collection and analysis was added in the model to test for association with biomarker levels. Linear regression adjusted on age and sex was used to explore the association between CSF and plasma NRG1. A receiver operator curve (ROC) analysis was performed to study the accuracy of plasma NRG1 in differentiating the different groups. The association of plasma NRG1 with core AD CSF biomarkers and CSF synaptic markers and with MMSE was explored by linear regression adjusted on age and sex in the whole cohort and in regard to Aβ status.

A *p-value* (*P*) < 0.05 was overall considered significant. Statistical analyses were performed using SPSS IBM 27.0 (IBM, Armonk, NY) and GraphPad Prism 9 (GraphPad Software, San Diego, CA, USA).

### Data availability

The datasets analyzed during the current study are available from the corresponding authors on a reasonable request.

## Results

### Cohort

Demographics and biomarker values in our cohort are reported in Table 1. A detailed description of non-AD dementia patients is reported in Supplemental Table 1. AD-MCI, AD dementia, and non-AD dementia patients were older than NC and non-AD MCI patients (*P* = 0.003 – *P* < 0.001). There was no difference regarding sex between groups (*P* = 0.155). All further analysis was adjusted on age and sex, unless otherwise specified. AD-MCI and AD dementia patients displayed decreased CSF Aβ42/Aβ40 ratio and increased p-tau and t-tau levels compared with other groups. CSF synaptic markers neurogranin, GAP-43, and SNAP-25 were significantly higher in AD patients compared to NC and displayed high accuracies in identifying AD (Supplemental Fig. 1). AD-MCI, non-AD MCI, AD dementia, and non-AD dementia patients had decreased MMSE compared to NC. Delay between the collection of samples and analysis was not associated with biomarker levels in uni- and multivariate analysis (results not presented); thus, it was not further added as a covariate.

**Table 1:** Demographics and biomarker values

***n*** = 127	Neurological controls *n* = 20	Non-AD MCI *n* = 19	AD-MCI *n* = 25	AD dementia *n* = 37	Non-AD dementia *n* = 26	***P-value***
Age, years	60.6 (9.6)	61.1 (8.4)	70.3 (5.8)^#^	67.7 (7.9)^#^	68.1 (7.0)^#^	**<0.001**
Female, *n* (%)	70% (14)	63% (12)	68% (17)	62% (23)	38% (10)	0.155
LoE, years	11.6 (3.8)	9.7 (2.5)	11.0 (3.9)	9.0 (3.5)^#^	11.2 (3.4)	**0.050**
MMSE	27.42 (1.6)	25.0 (2.3)^#^	25.1 (2.4)^#^	18.2 (4.3)^#^	22.8 (5.4)^#^	**<0.001**
**CSF biomarkers**						
CSF Aβ42, pg/mL	1041.6 (264.4)	987.2 (326.0)	516.4 (122.5)^#^	548.3 (135.9)^#^	919.3 (428.7)	**<0.001**
CSF Aβ42/Aβ40 ratio	0.129 (0.045)	0.092 (0.029)	0.051 (0.027)^#^	0.045 (0.017)^#^	0.104 (0.033)	**<0.001**
CSF p-tau, pg/mL	33.7 (10.7)	40.7 (17.2)	79.2 (22.9)^#^	95.7 (32.5)^#^	45.2 (20.5)^#^	**<0.001**
CSF t-tau, pg/mL	196.0 (66.7)	223.0 (100.6)	501.7 (203.4)^#^	703.9 (285.2)^#^	305.2 (152.1)^#^	**<0.001**
CSF NRG1, pg/mL	295.8 (107.1)	324.4 (137.5)	312.8 (157.8)	403.9 (155.1)^#^	315.0 (134.4)	**0.044**
CSF neurogranin, pg/mL	208.1 (69.5)	213.6 (84.7)	364.4 (83.1)^#^	351.4 (91.9)^#^	230.2 (107.9)	**<0.001**
CSF GAP-43, pg/mL	1677.2 (616.2)	2004.8 (987.9)	3422.9 (1087.9)^#^	3787.6 (1388.7)^#^	2348.8 (1267.8)^#^	**<0.001**
CSF SNAP-25, pg/mL	6.7 (2.2)	8.2 (3.5)	13.1 (3.7)^#^	17.1 (5.3)^#^	7.8 (3.9)	**<0.001**
**Plasma biomarker**						
Plasma NRG1, pg/mL	378.9 (400.7)	488.4 (392.2)	707.6 (562.7)^#^	940.3 (737.5)^#^	615.5 (486.3)^#^	**<0.001**

Data are shown as mean (SD) or *n* (%), as appropriate. The Kruskal-Wallis test was used to compare age between groups and Pearson’s chi-square to compare sex. Fluid biomarker levels and MMSE were compared with a one-way ANCOVA adjusted by age and sex followed by the least square difference test adjusted for multiple comparisons

*Abbreviations*: *Aβ42* amyloid-beta 42, *Aβ40* amyloid-beta 40, *AD* Alzheimer’s disease, *AD-MCI* MCI due to Alzheimer’s disease, *CSF* cerebrospinal fluid, *GAP-43* growth-associated protein 43, *LoE* level of education, *MCI* mild cognitive impairment, *MMSE* Mini-Mental State Examination, *NRG1* neuregulin 1, *p-tau* phosphorylated tau, *SNAP-25* synaptosomal-associated protein 25, *t-tau* total tau

^#^*P* < 0.05 compared to neurological controls

### Plasma NRG1 levels across groups

**Fig. 1:**
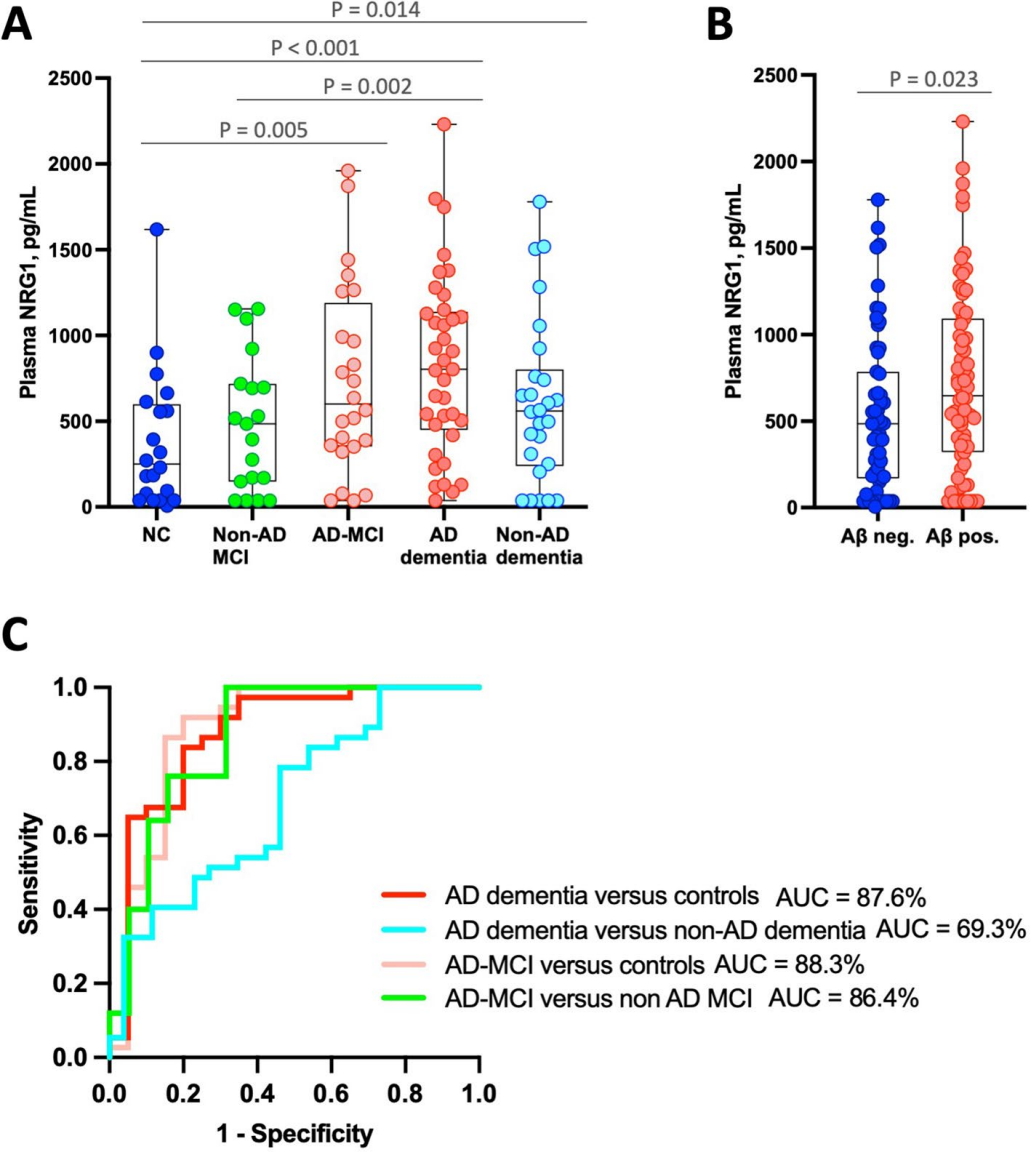
Plasma NRG1 levels across groups and correlation to CSF NRG1. **A** Box plots displaying plasma NRG1 levels across diagnosis groups. Levels were compared using a one-way ANCOVA adjusted on age and sex followed by a post hoc LSD test adjusted for multiple comparisons. **B** Box plot displaying plasma NRG1 levels in Aβ-positive patients (*n* = 62) and Aβ-negative patients (*n* = 55). Levels were compared using a one-way ANCOVA adjusted on age and sex followed by a post hoc LSD test adjusted for multiple comparisons. **C** ROC curves displaying plasma NRG1 accuracy in differentiating AD patients from neurological controls (AUC = 87.6%, 95% CI: 76.9–98.2%), AD dementia from non-AD dementia patients (AUC = 69.3%, 95% CI: 55.7– 82.3%), AD-MCI from NC (AUC = 88.3%, 95% CI: 77.2–0.99.6%), and AD-MCI from non-AD MCI patients (AUC = 86.4%, 95% CI: 74.7–98.3%). Abbreviations: Aβ, amyloid-beta; AD, Alzheimer’s disease; AUC, area under the curve; MCI mild cognitive impairment; LSD test, least square difference test; MCI, mild cognitive impairment; MMSE, Mini-Mental State Examination; NRG1, neuregulin 1; ROC, receiver operator characteristics

A higher concentration of plasma NRG1 was found in AD dementia patients compared to NC after adjustment on age and sex (940.3 versus 378.9 pg/mL, *P* < 0.001, Fig. 1A). AD-MCI patients also displayed higher concentrations compared to NC (707.6 versus 378.9 pg/mL, *P* = 0.005). Non-AD dementia had higher levels compared to NC (615.5 versus 378.9 pg/mL, *P* = 0.014). Plasma NRG1 concentration did not differ significantly between NC and non-AD MCI patients. Participants were then dichotomized according to their Aβ status defined by CSF Aβ42/Aβ40 ratio (Supplemental Table 2). Aβ-positive patients displayed higher plasma NRG1 concentration than Aβ-negative patients (774.0 versus 538.4 pg/mL, *P* = 0.023, Fig. 1B).

### Plasma NRG1 accuracy in identifying AD

We studied plasma NRG1 accuracy in discriminating AD patients from other diagnosis groups (Fig. 1C). Plasma NRG1 showed good performance in differentiating AD patients from NC both at MCI stage (AUC = 88.3%, 95% CI: 77.2–0.99.6%) and at dementia stage (AUC = 87.6%, 95% CI: 76.9–98.2%). When comparing AD-MCI to non-AD MCI patients, plasma NRG1 showed similar accuracy (AUC = 86.4%, 95% CI: 74.7–98.3%). However, its discriminating power was lower between AD patients and non-AD dementia patients (AUC = 69.3%, 95% CI: 55.7–82.3%).

### Correlation to CSF NRG1

**Fig. 2:**
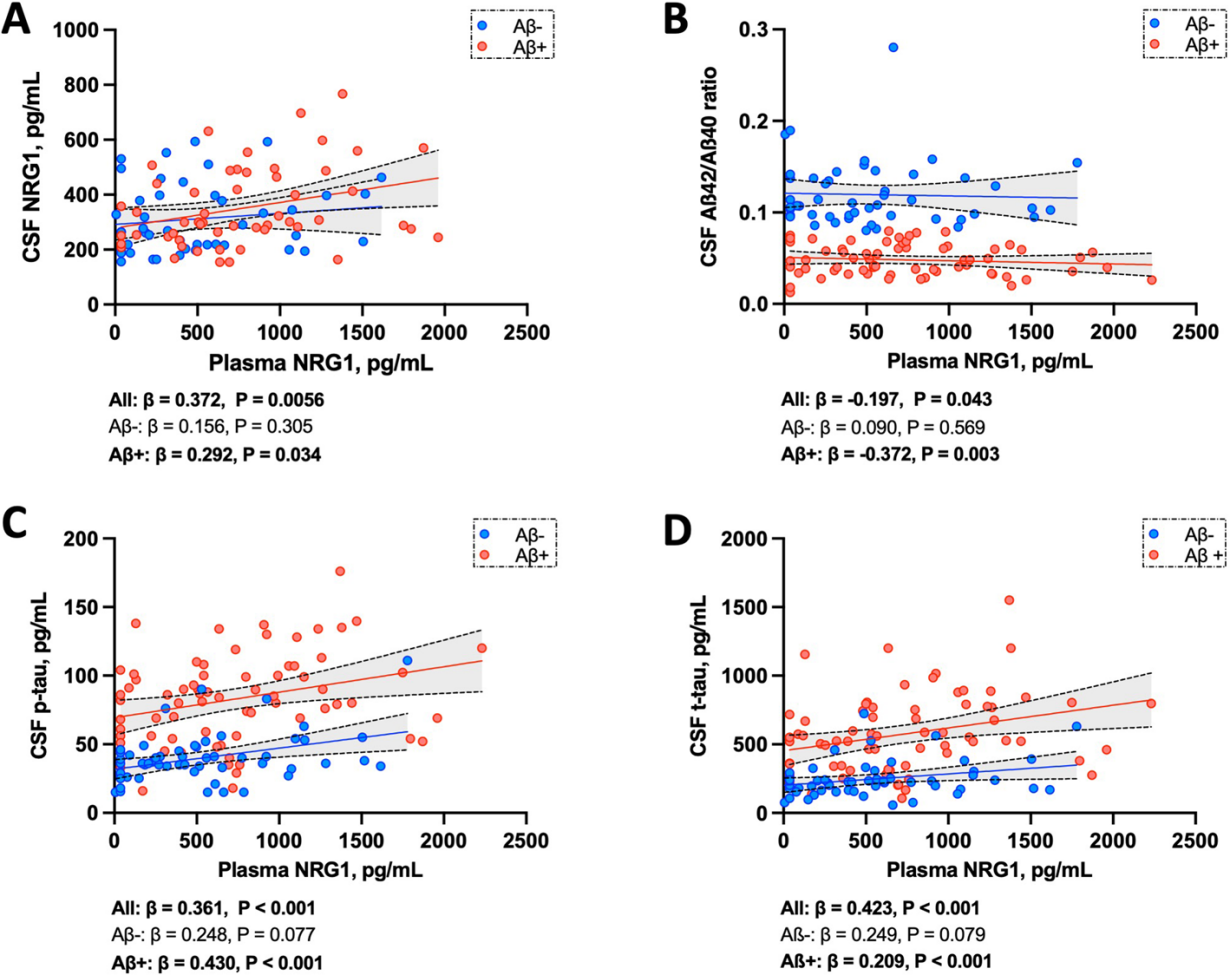
Association to AD CSF biomarkers. Association of plasma NRG1 with **A** CSF NRG1 **B** CSF Aβ42/Aβ40 ratio, **C** CSF p-tau, and **D** CSF t-tau, studied using linear regression adjusted on age and sex, in the whole cohort and in regard to Aβ status. Solid lines indicate the regression line and dashed lines, the 95% CI in Aβ-positive and Aβ-negative groups. Abbreviations: Aβ+, amyloid-beta positive; Aβ-, amyloid-beta negative; Aβ42, β-amyloid 42; Aβ40, β-amyloid 40; AD, Alzheimer’s disease; 95% CI, confidence interval; CSF, cerebrospinal fluid; NRG1, neuregulin 1; p-tau, phosphorylated tau; t-tau, total tau

Plasma and CSF NRG1 concentrations correlated in the overall cohort (*β* = 0.372, *P* = 0.0056, adjusted on age and sex) (Fig. 2A). This correlation was also detected in the Aβ-positive group (*β* = 0.292, *P* = 0.034). No correlation was observed between plasma and CSF NRG1 in the Aβ-negative group (*β* = 0.156, *P* = 0.305).

### Correlation to AD biomarkers

Plasma NRG1 displayed a weak inverse correlation with CSF Aβ42/Aβ40 ratio in the whole cohort (*β* = −0.197, *P* = 0.043, adjusted on age and sex, Fig. 2B). This correlation was stronger in the Aβ-positive group (*β* = −0.372, *P* = 0.003). CSF p-tau and CSF t-tau displayed a stronger correlation with plasma NRG1 in the whole population (respectively: *β* = 0.361, *P* < 0.001 and *β* = 0.423, *P* < 0.001) (Fig. 2C, D). These correlations were both sustained in the Aβ-positive patients (CSF p-tau: *β* = 0.430, *P* < 0.001; CSF t-tau: *β* = 0.209, *P* < 0.001). There was no correlation between plasma NRG1 and CSF Aβ42/Aβ40 ratio, p-tau, and t-tau in Aβ-negative patients.

### Association to synaptic biomarkers and to cognition

**Fig. 3:**
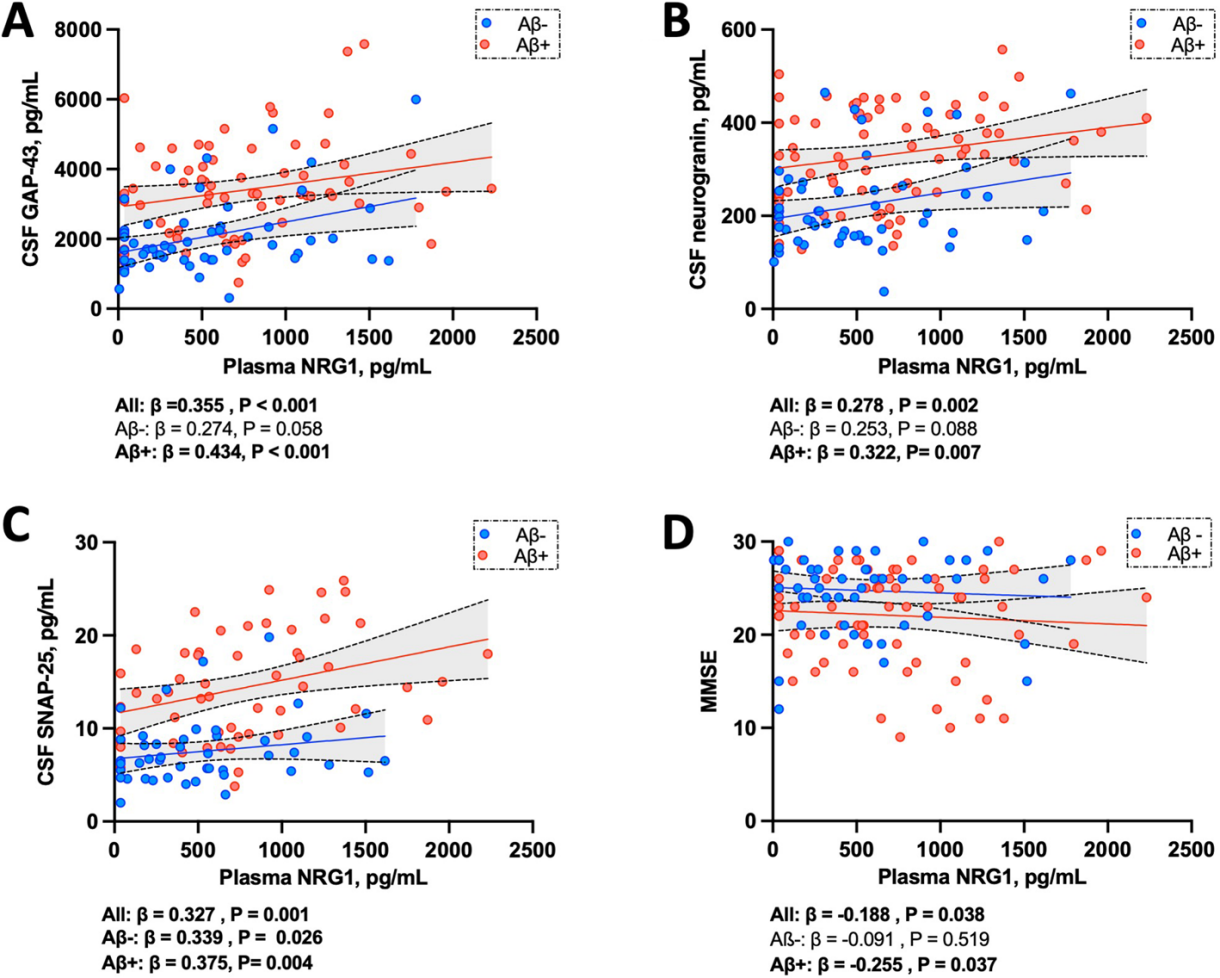
Association to CSF synaptic biomarkers and cognition. Association of plasma NRG1 with **A** CSF GAP-43, **B** CSF neurogranin, **C** CSF SNAP-25, and **D** MMSE, studied using linear regression adjusted on age and sex, in the whole cohort and in regard to Aβ status. Solid lines indicate the regression line and dashed lines, the 95% CI in Aβ-positive and Aβ-negative groups. Abbreviations: Aβ+, amyloid-beta positive; Aβ-, amyloid-beta negative; 95% CI, 95% confidence interval; CSF, cerebrospinal fluid; GAP-43, growth-associated protein 43; MMSE, Mini-Mental State Examination; NRG1, neuregulin 1; SNAP-25, synaptosomal associated protein 25

We studied the association of plasma NRG1 with three CSF synaptic biomarkers, neurogranin, GAP-43, and SNAP-25, after adjustment on age and sex (Fig. 3). Plasma NRG1 levels were overall associated with CSF GAP-43 levels (*β* = 0.355, *P* < 0.001) (Fig. 3A). This association remained significant in Aβ-positive patients (*β* = 0.434, *P* < 0.001) but not in the Aβ-negative group. Similarly, plasma NRG1 levels were associated with CSF neurogranin levels, in the whole cohort (*β* = 0.278, *P* = 0.002) and in the Aβ-positive patients (*β* = 0.322, *P* = 0.007) (Fig. 3B). CSF SNAP-25 levels were associated with plasma NRG1 in the whole cohort (*β* = 0.327, *P* = 0.001) as in the Aβ-positive (*β* = 0.375, *P* = 0.004) and in Aβ-negative group (*β* = 0.339, *P* = 0.026, Fig. 3C).

MMSE scores were significantly associated with plasma NRG1 levels after adjustment on age and sex (*β* = −0.188, *P* = 0.038, Fig. 3D). This association was sustained in the Aβ-positive group (*β* = −0.255, *P* = 0.037) but not in the Aβ-negative group.

## Discussion

Accessible biomarker monitoring synaptic dysfunction and loss would be of great clinical use in AD for early diagnosis, prediction, and monitoring of cognitive decline and for drug evaluation. In this study, we report that plasma synaptic marker NRG1 (i) was increased in AD patients already at the MCI stage; (ii) had a promising AUC to discriminate AD patients both at MCI and dementia stage, from NC; (iii) was associated with CSF AD biomarkers in Aβ-positive individuals; (iv) correlated with CSF synaptic markers; and (v) was inversely correlated with cognition.

NRG1 is expressed at the synapse in multiple brain regions, including those preferentially affected in AD, as the hippocampus and entorhinal cortex [32–34]. Post-mortem studies have reported NRG1 accumulation in neuritic plaques in association with dystrophic neurites, activated astrocytes, and microglia in human AD brains [21, 22]. NRG1- and ErbB4-directed immunoreactivity was observed in the hippocampus located in neuronal cell bodies and dendrites [22]. Interestingly, NRG1 can be measured in human fluids [17, 23, 24, 30, 31, 35, 36]. Increased levels of CSF NRG1 in AD compared with controls and with non-AD-related cognitive decline have been reported in the literature, including our prior work [23, 24]. In Pankonin et al., CSF NRG1 was increased in AD patients from an early stage of the disease. More recently, in a larger cohort using the most recent AD diagnosis criteria including CSF biomarkers, we have confirmed those results [24]. CSF NRG1 was significantly associated with CSF AD core biomarkers, suggesting a possible implication in AD pathophysiological processes. Moreover, CSF NRG1 levels correlated with other CSF synaptic markers, also suggesting that NRG1 was mainly originating from the synapse.

A previous study has already reported increased levels of plasma NRG1 in AD patients, with higher levels in advanced disease [35]. However, in this work, AD was clinically diagnosed with no biomarker to confirm the underlying AD pathophysiological process and correlation with CSF NRG1 levels was not studied. Our study brings evidence that plasma NRG1 is increased in patients with confirmed underlying amyloid pathology, already at the MCI stage. It is interesting to note as APP, at the origin of Aβ, and NRG1 are both cleaved by BACE1 in the brain [18, 20].

Plasma NRG1 levels were significantly correlated with CSF levels in the whole cohort and this association was sustained in the Aβ-positive patient group. The existence of extracerebral expression of NRG1 is known but the significant correlation between plasma and CSF levels indicates that plasma level modifications substantially arise from the central nervous system [37]. Thus, this flags plasma NRG1 levels as a potential surrogate for brain NRG1 modifications in AD. Our cohort was phenotyped using the measure of validated CSF biomarkers: GAP-43, neurogranin, and SNAP-25. Consistently with the existing literature, CSF synaptic biomarker levels were found to be altered in the AD group at MCI and dementia stages and they displayed interesting performance in separating the AD group from the control group. Significant correlation of plasma NRG1 with CSF synaptic markers in the Aβ-positive patients also supports that detected NRG1 changes are related to synaptic modifications.

There was a significant association between plasma NRG1 levels and MMSE in our whole cohort as well as in the Aβ-positive patients. This finding is in agreement with the previous studies in plasma and CSF again showing that NRG1 levels associate with cognition already at early stages of the disease [23, 24, 35].

Plasma NRG1 also displayed increased levels in non-AD dementia compared to NC and its accuracy in identifying AD at the dementia stage was moderate. In a study on vascular dementia, plasma NRG1 levels were found to be increased and inversely correlated to cognitive severity [38]. Neuropathological studies and synaptic CSF biomarker results have highlighted the fact that synapse dysfunction is a prominent feature in AD but that it is not entirely specific to it [39, 40]. It can also be observed in non-AD dementia, although to a much lesser extent than in AD, a finding in line with our results [41].

An underlying mechanistic question to this marker is whether alterations in NRG1 levels are related to a general process of synaptic degeneration and clearance or whether these changes occur as a response, positive or negative, to the development of AD pathology or to an increase in synaptic synthesis and release.

NRG1-ErbB4 signaling is important in regulating synaptic function at both excitatory and inhibitory synapses in the adult brain under physiological conditions [42, 43]. NRG1–ErbB4 signaling appears implicated in short-term synaptic plasticity through modulation of glutamatergic transmission. ErbB4 co-localizes and interacts with PSD95, a postsynaptic scaffold protein essential for the assembly and function of glutamatergic synapses [44]. Studies have shown that the pair can both suppress the induction and the expression of LTP [45, 46]. However, the NRG1 effect on neurotransmission might vary between brain regions. NRG1 administration decreased NMDA-receptor-mediated excitatory postsynaptic potentials in slices of the prefrontal cortex [47]. NRG1 decreased synaptic transmission in entorhinal CA1 but increased in response to entorhinal cortical stimulation in rats [48]. The levels of NRG1–ErbB4 signaling also impact GABAergic transmission and regulate signal integration by pyramidal neurons [43]. There is also evidence to support that NRG1 regulates long-term plasticity in the brain and NRG1 has been shown to stimulate the expression of receptors for key neurotransmitters, including glutamate, GABA, and ACh [49, 50]. Finally, NRG1–ErbB4 pathway is also implicated in neuron survival in different cellular populations including cortical neurons, dopaminergic neurons, motor neurons, and cochlear sensory neurons [42]. NRG1 was first identified as a major susceptibility gene in schizophrenia [51, 52]. Mutant NRG1 mice display both excitatory and inhibitory synaptic impairment and schizophrenia-like behavioral disorder [53]. While loss of NRG1 signaling has been shown to be pejorative to synaptic transmission, excessive NRG1 activity is also associated with synaptic dysfunction resulting from alteration of LTP at glutamatergic synapses [34, 54]. In line with those findings, evidence suggests that NRG1 increase may specifically influence cognitive function and neuropathology in AD [55–57]. Although not formally established, the mechanism of the increase of NRG1 could be explained by the increased levels and activity of BACE1 observed in AD [55]. Yet, its beneficial or detrimental effect is not solved. In experimental works, NRG1 overexpression could rescue APP-induced toxicity in primary cortical neurons [56]. In an AD mouse model, NRG1 treatment prevented amyloid β-induced impairment of long-term potentiation in hippocampal slices via its receptor ErbB4 [58]. Conversely, other experimental works have suggested a negative effect of the NRG1-ErbB4 signaling in AD. Perfusion of NRG1 in the hippocampus decreased LTP in the AD mouse model as well as in control mice [59]. Further understandings of NRG1 response upon amyloid pathology will allow to specify the exact synaptic events associated with CSF and plasma NRG1 modifications observed in AD patients.

In addition to contributing to better understanding of AD mechanisms, our finding that plasma NRG1 levels could reflect synaptic impairment in AD may have major practical utility. The development of blood biomarkers measuring Aβ, tau, and neurodegeneration processes has known great advancement recently, but, to date, there are no validated blood biomarkers reflecting synaptic pathology [13]. Recent studies have reported that the measure of markers of AD hallmarks in plasma such as Aβ42, p-tau 181, p-tau217, and p-231 can identify and monitor AD brain pathology with high accuracy, demonstrating that they can be used as non-invasive tools in AD diagnosis [60–62]. As synaptic impairment is one of the earliest abnormal features in AD, already present at the preclinical phase, an accessible non-invasive synaptic marker would be of high added value for early diagnosis [12].

Moreover, synaptic markers hold important promise for monitoring the effects of disease-modifying treatments on synaptic degeneration. Compared with CSF markers, validated blood-based synaptic AD biomarkers would provide a fast, acceptable, and cost-effective method of early detection, diagnosis, and follow-up as well as a screening and follow-up tool in therapeutic trials. Our work shows that plasma NRG1 levels could be one of these potential biomarkers.

### Limitations

This study has several limitations. The correlation between plasma and CSF NRG1 remained moderate. Further studies will be needed to understand if this variability is related to the blood-brain barrier’s passage, NRG1 metabolism in plasma, matrix effects, or interaction with peripheral NRG1. In our cohort, cognition was evaluated using MMSE, a general test. A study of plasma NRG1 relation to cognition using neuropsychological assessment with tests evaluating specifically episodic memory should give more robust evidence. We could not include measurements of AD-specific blood biomarkers such as p-tau or Aβ. Finally, confirmation of our results in larger cohorts is needed, including larger samples of non-AD dementia patients. A study of plasma NRG1 at the preclinical phase will also be needed to better characterize its kinetic on the whole AD spectrum.

## Conclusion

Our results suggest that plasma NRG1 is a novel biomarker for synaptic dysfunction/degeneration in AD. Plasma NRG1 showed a significant increase in AD patients already at the MCI stage and correlated with biomarkers for AD pathology, as well as with established CSF biomarkers for synaptic dysfunction in AD. As a novel blood synaptic marker, plasma NRG1 may improve the diagnosis of neurodegenerative disorders and may also be useful to monitor clinical disease progression and therapeutic response in clinical trials of novel disease-modifying drug candidates.

## Supplementary Information

**Additional file 1: Supplementary Table 1.** Demographics and biomarkers values of patients with non-AD dementia subgroups. **Supplementary Table 2.** Demographics and biomarkers values according to Aβ status. **Supplementary Figure 1.** CSF synaptic markers levels across groups and diagnostic accuracies.

## Data Availability

The datasets analyzed during the current study are available from the corresponding authors on a reasonable request.

## Abbreviations

AD: Alzheimer’s disease
Aβ40: Amyloid beta 1–40
Aβ42: Amyloid beta 1–42
APP: Amyloid precursor protein
AUC: Area under the curve
BACE1: Beta-site amyloid precursor protein cleaving enzyme 1
CSF: Cerebrospinal fluid
erbB4: Receptor tyrosine-protein kinase erbB-4
GAP-43: Growth-associated protein 43
MCI: Mild cognitive impairment
MMSE: Mini-Mental State Examination
NRG1: Neuregulin 1
PET: Positron emission tomography
p-tau: Phosphorylated tau
SNAP-25: Synaptosomal associated protein 25
t-tau: Total tau
