# Supplementary material for: Plasma neuregulin 1 as a synaptic biomarker in Alzheimer’s disease: a discovery cohort study
Source: Alzheimers Res Ther. 2022 May 23;14:71. doi: 10.1186/s13195-022-01014-7 (PMC9125890; doi:10.1186/s13195-022-01014-7)
Supplement: Supplementary file 1 — Additional file 1: Supplementary Table 1. Demographics and biomarkers values of patients with non-AD dementia subgroups. Supplementary Table 2. Demographics and biomarkers values according to Aβ status. Supplementary Figure 1. CSF synaptic markers levels across groups and diagnostic accuracies. [file 13195_2022_1014_MOESM1_ESM.docx]

**SUPPLEMENTARY DATA**

**Supplementary Table 1, Demographics and biomarkers values of patients with non-AD dementia subgroups**

**Supplementary Table 2, Demographics and biomarkers values according to Aβ status**

**Supplementary Figure 1, CSF synaptic markers levels across groups and diagnostic accuracies**

**Supplementary Table 1, Demographics and biomarkers values of patients with non-AD dementia subgroups**

|  | **Neurological controls** | **Non-AD MCI** | **AD-MCI** | **AD dementia** | **DLB** | **FTD** | **VaD** | **Other non-AD dementia*** |
| --- | --- | --- | --- | --- | --- | --- | --- | --- |
|  | *n* = 20 | *n* = 19 | *n* = 25 | *n* = 37 | *n* = 6 | *n* = 9 | *n* = 5 | *n* = 6 |
| Age, years | 60.6 (9.6) | 61.11 (8.4) | 70.3 (5.8) | 67.7 (7.9) | 67.2 (6.9) | 65.9 (7.2) | 70.2 (6.3) | 70.5 (7.6) |
| Female, % (n) | 14 (70%) | 12 (63%) | 68% (17) | 62% (23) | 33 % (2) | 33 % (3) | 20% (1) | 67% (4) |
| Levels of education, years | 11.6 (3.8) | 9.7 (2.5) | 11.0 (3.9) | 9.0 (3.5) | 11.7 (4.1) | 10.7 (3.6) | 10.3 (5.0) | 11.8 (1.9) |
| MMSE | 27.4 (1.6) | 25.06 (2.4) | 25.1 (2.4) | 18.2 (4.3) | 21.5 (6.9) | 23.0 (5.8) | 22.4 (5.3) | 24.0 (4.6) |
| **CSF biomarkers** |  |  |  |  |  |  |  |  |
| CSF Aβ42, pg/mL | 1041.6 (264.4) | 987.16 (326.0) | 516.4 (122.5) | 548.3 (135.9) | 838.5 (237.6) | 1030.4 (645.9) | 803.8 (324.4) | 929.7 (259.6) |
| CSF Aβ42/Aß40 ratio | 0.129 (0.045) | 0.092 (0.029) | 0.051 (0.0275) | 0.045 (0.017) | 0.091 (0.025) | 0.113 (0.051) | 0.102 (0.009) | 0.103 (0.017) |
| CSF p-tau, pg/mL | 33.7 (10.7) | 40.69 (17.2) | 79.2 (22.9) | 95.7 (32.5) | 48.2 (19.8) | 50.5 (25.0) | 36.0 (13.2) | 41.8 (20.0) |
| CSF t-tau, pg/mL | 196.0 (66.7) | 223.00 (100.6) | 501.7 (203.4) | 703.9 (285.2) | 287.3 (143.6) | 363.0 (197.1) | 257.2 (122.7) | 276.5 (105.2) |
| CSF NRG1, pg/mL | 295.8 (107.1) | 324.37 (137.5) | 312.8 (157.8) | 403.9 (155.1) | 299. 6 (162.2) | 288.3 (115.6) | 268.4 (93.6) | 388.1 (147.3) |
| CSF neurogranin, pg/mL | 208.1 (69.5) | 213.61 (84.7) | 364.4 (83.1) | 351.4 (91.9) | 216.0 (104.0) | 264.5 (113.9) | 180.0 (80.0) | 235.4 (133.6) |
| CSF GAP-43, pg/mL | 1677.2 (616.2) | 2004.8 (987.9) | 3422.9 (1087.9) | 3787.6 (1388.7) | 2238.0 (1452.7) | 2749.3 (1490.6) | 1997.3 (858.3) | 2112.6 (1105.6) |
| CSF SNAP-25, pg/mL | 6.7 (2.2) | 8.2 (3.5) | 13.1 (3.7) | 17.1 (5.3) | 9.73 (5.9) | 7.60 (2.2) | 6.12 (3.5) | 7.75 (4.0) |
| **Plasma biomarker** |  |  |  |  |  |  |  |  |
| Plasma NRG1, pg/mL | 378.9 (400.7) | 488.4 (392.2) | 707.6 (562.7)^#^ | 940.3 (737.5)^#^ | 710.99 (274.5) | 479.93 (565.7) | 784.2 (701.1) | 582.7 (353.8) |

Data shown as mean (SD) or n (%), as appropriate. *Other dementia on-AD dementia group included patients with alcohol related dementia (n=2), Huntington disease (n=1), and dementia of unknown cause (n=3).

Abbreviations: Aβ42, β-amyloid 42; Aβ40, β-amyloid 40; AD, Alzheimer’s Disease; CSF, cerebrospinal fluid; MCI mild cognitive impairment; MMSE, Mini-Mental State Examination; NRG1, Neuregulin-1; p-tau, phosphorylated tau; t-tau, total tau.

**Supplementary Table 2, Demographics and biomarkers values according to Aβ status**

|  | **Aβ-negative participants** | **Aβ-positive participants** | **P-value** |
| --- | --- | --- | --- |
|  | *n* = 65 | *n* = 62 |  |
| Age, years | 63.7 (9.2) | 68.1 (7.4) | **0.002** |
| Female, % (n) | 55% (36) | 65% (40) | 0.366 |
| Levels of education, years | 11.4 (3.3) | 9.7 (3.7) | **0.025** |
| MMSE | 24.9 (4.1) | 22.0 (5.4) | **0.005** |
| **CSF biomarkers** |  |  |  |
| CSF Aβ42, pg/mL | 1013.9 (360.1) | 568.3 (169.5) | **<0.001** |
| CSF Aβ42/Aβ40 ratio | 0.119 (0.035) | 0.048 (0.017) | **<0.001** |
| CSF p-tau, pg/mL | 40.1 (18.3) | 82.4 (32.6) | **<0.001** |
| CSF t-tau, pg/mL | 246.7 (131.6) | 570.9 (285.1) | **<0.001** |
| CSF NRG1, pg/mL | 312.0 (125.6) | 359.6 (157.1) | **0.05** |
| CSF neurogranin, pg/mL | 223.2 (95.4) | 331.9 (101.7) | **<0.001** |
| CSF GAP-43, pg/mL | 2076.0 (1091.5) | 3382.7 (1374.5) | **<0.001** |
| CSF SNAP-25, pg/mL | 7.5 (3.5) | 14.3 (5.5) | **<0.001** |
| **Plasma biomarker** |  |  |  |
| Plasma NRG1, pg/mL | 538.4 (460.9) | 774.0 (659.1) | **0.023** |

Data shown as mean (SD) or n (%), as appropriate. Mann-Whitney test was used to compare age between groups and Pearson’s chi-square to compare sex. Fluid biomarkers levels and MMSE were compared with a one-way ANCOVA adjusted by age and sex.^#^P < 0.05 indicates significance.

Abbreviations: Aβ42, β-amyloid 42; Aβ40, β-amyloid 40; AD, Alzheimer’s disease; AD MCI, MCI due to Alzheimer's disease; CSF, cerebrospinal fluid; GAP-43, growth-associated protein 43; LoE, level of education; MCI mild cognitive impairment; MMSE, Mini-Mental State Examination; NRG1, Neuregulin-1; p-tau, phosphorylated tau; SNAP, synaptosomal-associated protein 25; t-tau, total tau.

**Supplementary Figure 1, CSF synaptic markers levels across groups and diagnostic accuracies**

**A,C,E** Box-plots comparing: **A,** CSF neurogranin levels; **C,** CSF GAP-43 levels; **E,** CSF SNAP-25 levels across clinical groups. The box-plots depict the median (horizontal bar), interquartile range (IQR, hinges) and 1.5 x IQR (whiskers). Group comparisons were computed with a one-way ANCOVA adjusting for age and sex. Least square difference (LSD) test was used for the post hoc pairwise comparisons an Bonferroni was used to adjust for multiple comparisons. P < 0.05 indicates significance.

**B, D,E** ROC curves displaying **B,** CSF neurogranin levels; **D,** CSF GAP-43 levels; **F,** CSF SNAP-25 levels accuracies in differentiating AD-MCI from NC, AD-MCI from non-AD MCI, AD dementia from NC and AD dementia from non-AD dementia.

Abbreviations: AD, Alzheimer’s disease; AUC, area under the curve; CSF, cerebrospinal fluid; MCI, mild cognitive impairment; NC, neurological controls; ROC, receiver operator characteristic.
